# Epidermal Development in Mammals: Key Regulators, Signals from Beneath, and Stem Cells

**DOI:** 10.3390/ijms140610869

**Published:** 2013-05-24

**Authors:** Shuang Liu, Huishan Zhang, Enkui Duan

**Affiliations:** 1 State Key Laboratory of Reproductive Biology, Institute of Zoology, Chinese Academy of Sciences, Beijing 100101, China; E-Mails: liushuang@ioz.ac.cn (S.L.); zhanghs@ioz.ac.cn (H.Z.); 2 University of Chinese Academy of Sciences, Beijing 100049, China

**Keywords:** epidermal development, hair follicles, epidermal stem cells, mesenchyme-epithelial interactions, pluripotent stem cells

## Abstract

Epidermis is one of the best-studied tissues in mammals that contain types of stem cells. Outstanding works in recent years have shed great light on behaviors of different epidermal stem cell populations in the homeostasis and regeneration of the epidermis as well as hair follicles. Also, the molecular mechanisms governing these stem cells are being elucidated, from genetic to epigenetic levels. Compared with the explicit knowledge about adult skin, embryonic development of the epidermis, especially the early period, still needs exploration. Furthermore, stem cells in the embryonic epidermis are largely unstudied or ambiguously depicted. In this review, we will summarize and discuss the process of embryonic epidermal development, with focuses on some key molecular regulators and the role of the sub-epidermal mesenchyme. We will also try to trace adult epidermal stem cell populations back to embryonic development. In addition, we will comment on *in vitro* derivation of epidermal lineages from ES cells and iPS cells.

## 1. Introduction

The mammalian skin epidermis together with its derivative appendages, such as hair follicles, sebaceous glands and sweat glands, plays pivotal functions in protecting the organism from dehydration and environmental insults, as well as in regulating the body temperature. The stratified epidermis and its appendages harbor niches for several types of stem cells and make an ideal model to study stem cell biology. To establish these delicate structures and functions, the epidermal development requires tight spatiotemporal regulation of interactions between the epidermis and its underlying mesenchyme, the dermis, which also contains types of stem/progenitor cells [1,2]. Comprehensive knowledge about adult epidermal homeostasis and regeneration, as well as adult epidermal stem cell biology, has been accumulated for decades. However, insights into the cellular and molecular events during epidermal development are gained only in recent years, along with the progress in fate mapping techniques and site- and time-specific gene targeting strategies. In the present review, we will discuss the latest understanding about epidermal development and epidermal stem cell populations, as well as the progress in deriving epidermal lineages from pluripotent stem cells *in vitro*.

## 2. An Overview of the Development of Mammalian Epidermis and Its Appendages

The vertebrate epidermis originates from embryonic surface ectoderm, which is a single layer of ectodermal cells covering the embryo after neuralation. The surface ectoderm is a simple epithelium consisting of flat cells that express the cytokeratins K8/K18 [3,4]. It should be noted that a transient protective layer of endodermis-like cells, called the periderm, forms overlaying the surface ectoderm, and it is destined to be shed off once the epidermis starts stratification.

Before stratification, around E9.5 in mouse embryos for instance, K5/K14 expression replaces K8/K18 expression, which marks the event called epidermal commitment [5]. Then surface ectoderm becomes the embryonic epidermal basal layer that gives rise to all the structures of the future epidermis. It is believed that the sub-ectodermal mesenchyme sends signals to initiate epidermal stratification. The basal layer cells proliferate and form an intermediate cell layer under the periderm [5,6]. The intermediate layer cells divide and mature into spinous cells expressing K1/K10, so the intermediate layer is then called the spinous layer [6]. Spinous cells continue to differentiate, mature and migrate outwards to form the granular layer and the cornified layer, successively. Granular layer expresses Involucrin and Transglutaminase and cornified layer expresses Filaggrin and Loricrin [7]. Finally, the cornified layer cells become flattened, form cornified envelopes consisting of keratin proteins, and acquire the barrier function. During postnatal life, dead cells of the cornified layer are constantly shed and renewed. The maintenance of epidermal homeostasis is dependent on basal layer cells.

Development of the epidermis also includes the morphogenesis of epidermal appendages such as hair follicles and sweat glands. The development of a mouse hair follicle is morphologically divided into 8 stages (summarized from [8]). In stage 0, the epidermal basal layer remains morphologically uniform. In stage 1, the basal cells form a visible hair placode which is the primordial of a hair follicle, and dermal fibroblasts begin to aggregate under the placode. In stage 2, the hair placode elongates towards the dermis while the underlying “cap” of dermal fibroblast condensate become more evident. In stage 3, the epidermal cells form a multi-layered and elongated column called the *hair peg*, and the dermal condensate becomes a spherical dermal papilla (DP) in adjacent to the *hair peg*. In stage 4, the *hair peg* thickens at the lower end to form a hair bulb and half encloses the elongated dermal papilla. A corn-shaped inner root sheath (IRS) forms above the dermal papilla. During stage 5, the IRS extends upward the hair follicle and the bulge becomes visible. The elliptical dermal papilla is almost completely enclosed and the first sebocytes occur. In stage 6, hair follicle down-growth reaches the subcutis and the IRS forms a hair shaft at the upper end. The dermal papilla becomes thinner and totally gets enclosed. The sebaceous gland forms at the upper part of the hair follicle. In stage 7, the tip of the hair shaft leaves the IRS and enters the hair canal, and the dermal papilla gets even thinner. Finally, in stage 8, the hair shaft protrudes from the skin surface and the hair follicle reaches its maximal length. The morphogenesis of a hair follicle requires intensive communications and joint development of the epidermal and dermal compartments which will be discussed in detail later.

A sebaceous gland (SG) usually locates at the upper part of a hair follicle and is an integral part of a philosebaceous unit that secretes sebum to lubricate the skin and keep the waterproof property of hair in mammals. In humans, sebaceous glands develop around Week 13–14 of gestation [9]. In mice, sebaceous glands develop near the end of embryogenesis (Stage 5 of hair follicle morphogenesis) and mature after birth [9]. Compared with the interfollicular epidermis (IFE) and the hair follicle, the sebaceous gland was neglected for years, but it has recently received great research interests. Progress has been made in the research of sebaceous gland development, especially regarding sebaceous gland stem/progenitor cells.

Sweat glands, the most abundant glandular structure of the human body [10], are also epidermal derivatives. They are surrounded by adipose tissues and closely related to nerve fibers. Eccrine sweat gland, which is the primary form of cooling in humans, contains of a single long sweat duct and an unbranched coiled sweat gland extending deep into the dermis. Both the sweat duct and the coiled gland consist of two cell layers. Although sweat glands are important for the thermoregulation in mammals, still little is known about the regulation of their homeostasis and development.

In the following sections, the process of epidermal development will be further dissected, from several angles: major signaling events and the interactions between the epidermis and its underlying mesenchyme, specification and behaviors of distinct epidermal stem cell populations, as well as *in vitro* recapitulation of epidermal lineage differentiation from pluripotent stem cells.

## 3. Key Signaling Events and Mesenchymal-Epithelial Interactions during the Development of the Epidermis and its Appendages

In mammals, epidermal development is a multistage process consisting of epidermal specification, commitment, stratification and terminal differentiation, as well as morphogenesis of its derivatives. During the whole process, distinct signaling patterns specify different developmental stages and these stage-specifically regulated signaling events ensure the correct morphogenesis of skin epidermis and its appendages. Furthermore, as in other epitheliums, every step of epidermal development is closely related to its underlying mesenchyme, the dermis. On one hand, mesenchymal signals guide the formation of epidermis and its appendages. Differences in the dermis result in the regional heterogeneities in the epidermis [11]. On the other hand, the reciprocal mesenchymal-epithelial interactions also greatly contribute to the development of the dermis itself. The joint development of epidermis and dermis requires a tightly controlled sequence of signaling events that involve both compartments. In this section, we will expound the major signaling events in a context of mesenchymal-epithelial interactions during these developmental processes.

### 3.1. Adoption of the Epidermal Fate

The epidermis originates from the embryonic ectoderm which also gives rise to the nervous system. The choice of ectodermal cells between epidermal and neural fates is made shortly after gastrulation, depending on the balanced effects of Wnt, FGF and BMP signaling. Without Wnt signals, the ectodermal cells respond to FGF signals which could inhibit BMP signaling activity and thus develop towards a neural fate [12]. With Wnt signals, in contrast, the ectodermal cells respond to BMP signaling instead of FGF signaling, which results in the adoption of an epidermal fate [13]. These ectodermal cells expressing K8/K18 constitute the surface ectoderm which gives rise to the future epidermis, mammary glands, corneal epithelium, and so forth. These findings greatly facilitate *in vitro* epidermal differentiation from pluripotent stem cells, which will be discussed later.

### 3.2. Commitment of the Surface Ectoderm to Stratification: Formation of the Embryonic Basal Layer

The onset of K5/K14 expression in surface ectodermal cells is a hallmark for epidermal commitment and the formation of the epidermal basal layer. Nearly twenty years ago, it was observed that K5 expression in mouse embryos could be first detected in the posterior somatic ectoderm at E9.5, when mesodermal cells were beginning to populate the skin. Then K5 expression started to expand anteriorly and posteriorly [5]. According to these findings, the authors believed that the mesodermal dermomyotome provided the inductive clues. In fact, later studies demonstrate that interactions between the surface ectoderm and the underlying mesenchyme occur much earlier than this time point.

Some independent studies demonstrate the pivotal roles of p63, a member of the p53 family, in epidermal commitment, K5/K14 induction, and the following epidermal stratification [14,15]. Also, p63 is crucial in the self-renewal of epidermal stem cells [16], which we will discuss in next section. P63 expression can be detected as early as E8.5 in mice, preceding any overt phenotypic change towards commitment ([7] and our unpublished data). It is believed that certain mesenchymal signals drive p63 expression in the ectoderm, although the nature of the signals has not yet been identified. P63 has two major isoforms, TAp63 and DNp63. Unlike the Koster group who are convinced that the TAp63 is the main isoform expressed in early epidermal morphogenesis [17,18], some other groups, including ours, detect DNp63, rather than TAp63, during this period [19]. We found that in E8.5 mouse embryos, DNp63 expression is clearly seen in surface ectodermal cells overlying the newly formed somites, while it is hardly detected in cells overlying the unsegmented mesoderm (unpublished data from our lab). According to the location of p63 expression, it is possible that only mesenchyme that has developed to a certain degree could produce the inductive signals for epidermal commitment.

Several p63 target genes have been identified to function in the initial establishment of epidermal basal layer. It is shown that DNp63 can directly induce K14 expression [20,21], while TAp63 can indirectly induce the expression of K14 through AP-2γ [17]. In addition, p63 can directly drive the expression of the desmosome component Perp, whose emergence is related to the acquisition of an epidermal fate [22], in the embryonic basal layer [23].

### 3.3. Stratification of the Epidermis

Knowledge about the epidermal stratification mostly comes from studies in postnatal mature skin where the epidermis constantly renews itself by the replenishment from the basal layer. Basal cells divide and differentiate while migrating outwards, and their progeny are finally shed from the skin surface. Embryonic epidermis and adult epidermis share some common mechanisms during stratification.

The establishment of embryonic basal layer is accompanied by the formation of the basement membrane which separates the dermis and epidermis and provides the epidermal basal cells with extracellular matrix (ECM) proteins and growth factors. To achieve differentiation and stratification, basal layer cells have to escape the underlying basement membrane. Basal cells attach to the basement membrane through hemidesmosomes and focal adhesions, both of which are composed of integrins [13]. Earlier *in vitro* studies about human keratinocytes suggested that the down-regulation of integrins results in the detachment of differentiated cells from the basement membrane, while stem cells maintaining high levels of integrins remain attached to the membrane and undifferentiated [24,25]. Nevertheless, recent *in vivo* studies in mouse embryos suggested another model: upon stratification, the basal cells change their mitotic spindle orientation from lateral to perpendicular to the membrane, generating a committed suprabasal cell and an undifferentiated basal cell [26]. This asymmetric division of basal cells seems to require p63 [26], which is expressed in the basal cells prior to the formation of intermediate layer [7]. Down-regulating DNp63 in epidermis disables the formation of the intermediate layer [27].

The intermediate layer is a transient layer only existing in embryonic skin. Cells in the intermediate layer divide and mature to generate the post-mitotic spinous cells, and are soon replaced by the latter. Both intermediate and spinous cells express K1, whose expression is induced by target genes of DNp63 [27]. Notch signaling is suggested to be one of downstream effectors of DNp63 in inducing K1 expression. Supporting that, Notch pathway is found active in the intermediate layer [28,29], and K1 is proven a Notch target in the epidermis [30]. Inhibiting Notch signaling in epidermis leads to reduced K1 expression [30], whereas over-activation of Notch signaling results in spinous layer expansion [31]. The maturation of intermediate cells into spinous cells requires IKKα, a DNp63 target gene that is crucial for the cell-cycle withdrawal of intermediate cells [27]. Knockout mouse models also revealed Ovol1 [32], IRF6 [33,34] and 14-3-3σ [35,36] as important regulators of spinous layer maturation, although relationships between these different regulators in this process still needs further exploration. It should be noted that the intermediate layer does not exist in postnatal skin, where the post-mitotic spinous cells are directly differentiated from proliferating basal cells. Nevertheless, Notch signaling is also critical in postnatal epidermis for the inhibition of basal cell proliferation and initiation of terminal differentiation [37].

The following formation of granular layer and the cornified layer is largely regulated by the increase in extracellular Ca^2+^ concentration and the corresponding intracellular Ca^2+^ sensing machinery (reviewed in [7]). It is also suggested that protein kinase C (PKC) plays a role in the transition from spinous cells to granular cells [7]. During the final step of epidermal development, transcription factors Klf-4 [38] and Grhl3 [39,40] play important roles in the establishment of skin barrier function.

**Figure 1:**
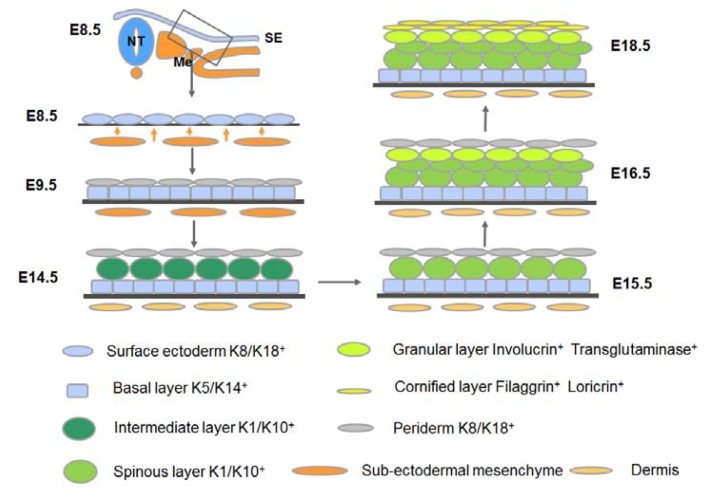
Development of stratified epidermis in mouse embryos. The epidermis is derived from embryonic surface ectoderm. After epidermal commitment, the surface ectoderm becomes the embryonic epidermal basal layer, which subsequently gives rise to the intermediate layer, spinous layer, granular layer and cornified layer. Different layers are labeled in different colors, with their specific markers annotated. NT, neural tube; Me, mesoderm; SE, surface ectoderm.

The commitment and stratification of mammalian epidermis is summarized in Figure 1.

### 3.4. Hair Follicle Morphogenesis

The morphogenesis of hair follicles is an excellent example of mesenchymal-epithelial interactions. The well-organized sequential events require tightly spatiotemporal regulation of signals transmitted between the dermis and the epidermis, resulting in the joint differentiation of both compartments.

#### 3.4.1. First Dermal Signal(s)

In mouse embryos, primary hair follicle placodes are formed at E14.5. These placodes are the first morphological sign for hair follicle development. Early recombination experiments indicated that the “first inductive signal(s)” for hair follicle placode initiation came from the dermis [41]. Although the nature of the first signal(s) remains elusive, with the help of genetic mouse models, it has been demonstrated that the Wnt/β-catenin pathway activation in the dermis is obligatory for the secretion of the first signal(s). Supporting that, dermal-specific deletion of β-catenin activity leads to the failure in placode initiation, while over-activated β-catenin results in enlarged placodes with accelerated follicle differentiation [42]. Interestingly, the activation of dermal β-catenin is driven by epidermis-derived Wnt ligands, which so far seem to be the most upstream signal for hair follicle initiation [42]. The expression of Wnt ligands in the epidermis is widespread, and the activation of β-catenin in the upper dermis is correspondingly uniform, suggesting that the first dermal signals are also uniform rather than specifically localized [43].

#### 3.4.2. Formation of Hair Placodes and Dermal Condensates

Preceding the morphological formation of hair placodes, the expression of some molecules becomes patterned in both epidermis and dermis. These marker molecules include epidermal Wnt10b, Edar, Dkk4 and K17, as well as dermal Sox2 and Sdc1 (reviewed in [44]). At this point, both hair follicle promoters and repressors are expressed, whose competition is believed to result in the formation of a regulated and patterned array of hair placodes, via a reaction-diffusion mechanism [43,45].

In mouse embryos, following dermal β-catenin activation [42], epidermal Wnt activity gradually increases in the pre-placode regions [42,46], earlier than other pre-placode markers [46]. Also, dermal Wnt activity becomes focused into regions that subsequently form the dermal condensates [42,47]. Such patterned Wnt activity in the dermis and the formation of dermal condensates require epidermal β-catenin activity: ablation of epidermal Wnt activity leads to failure in dermal condensate formation while its over-activation results in ectopic hair follicles [42,48]. Several Wnt inhibitors such as Dkk1 and Dkk4 are also dynamically and positionally expressed in the developing skin [45,49–51], presumably to balance the Wnt activity [43].

After the initial induction of the primary hair placodes, placode stabilization and patterning involve the epidermal Eda/Edar/NFκB pathway, whose activation lies downstream of Wnt signaling [52]. The TNF family ligand Ectodysplasin A (Eda) binds to its receptor Edar and induces nuclear translocation and transcriptional activation of transcription factor NFκB [53–55]. Both the ligand and receptor are only expressed in the epidermal compartment. Although initial activation of Wnt signaling in the epidermis precedes the Eda/Edar/NFκB signaling, the latter is responsible for the refinement of focal Wnt signaling within the placodes. Downstream effectors of Eda/Edar signaling include both placode promoters and inhibitors, such as epidermal Wnt ligand Wnt10b [46], Wnt inhibitor Dkk4 [51,56], BMP inhibitors Ccn2 and Ctgf, and Sonic hedgehog (Shh) [57], as well as dermal BMP4 and BMP7 [57–59]. Thus, this multi-facet pathway has a dual role in placode fate promotion and in the lateral inhibition of placode fate in surrounding cells [43].

BMP signaling activity is a major inhibitor of placode formation. BMPs such as BMP2, BMP4 and BMP7 are expressed intensively in the (pre-)placode or the dermal condensate [48,60,61]. It is suggested that BMPs can diffuse into interfollicular region to suppress the placode fate, while short-range-acting BMP inhibitors, such as Noggin, Follistatin, Ccn2 and Ctgf, are expressed in placode region to counteract BMPs and to ensure the follicular fate [43,44,57,61–64].

Other signals such as FGF signals and Notch signal have been implicated in the placode formation, but their functions require further study [43,44].

#### 3.4.3. Hair Follicle Down-Growth

After formation, the placode starts to grow down into the dermis following placode cell proliferation. The signals that guide the follicle down-growth are derived from the dermal condensates, that is, the developing dermal papilla.

One of the major signaling pathways that regulate the follicle down-growth is PDGF signaling. It is known for years that the platelet derived growth factor A (PDGFA) is expressed exclusively in the epidermis while its receptor PDGFRA is expressed in the dermis. Both the ligand and receptor are initially expressed broadly and then become focused in the placode and dermal condensate, respectively [65]. Knockout of both the ligand and receptor in mouse embryos does not prevent placode or dermal condensate formation, but severely blocks their further development [65]. It can be inferred that silencing PDGF signaling within the dermis leads to defects in the constitution of dermal papilla, which in turn impedes the dermal papilla-secreted factors that are responsible for the growth of epidermal components.

Like PDGF signaling, Shh signaling is not involved in placode or dermal condensate formation, but functions in subsequent steps. Expression of Shh in the placode relies on the aforementioned Wnt and Eda signaling. Shh expression gradually concentrates on the tip of the placode adjacent to the developing dermal papilla. Although Shh receptor Patched is expressed in both epidermal placodes and dermal condensates [65], only Shh activity in the dermal condensate is crucial for dermal papilla development and the following events [66]. Several Shh downstream targets within the dermal condensates have been suggested, such as TGF-β, Wnt5a and PDGFRA [50,65]. Shh regulates dermal PDGFRA expression and is therefore important for the dermal reactiveness to epidermis-secreted PDGFA.

TGF-β signaling, specifically TGF-β2, is also important for hair follicle growth. During the early stage of follicle down-growth, TGF-β2 is expressed in both the hair bud and the dermis, and Smad-2 is activated in the hair bud [67]. TGF-β2 transiently induces the transcription factor Snail and activates MAPK pathway in the hair bud, resulting in bud cell proliferation [67]. Hair follicle morphogenesis is profoundly delayed and hair follicle numbers are reduced by half in TGF-β2 null mice, while treatment with TGF-β2 protein induced hair follicle development in wildtype embryonic skin explant [68], indicating that TGF-β2 is both required and sufficient to promote hair follicle morphogenesis. It is also shown that TGF-β2 is specifically expressed in human dermal papilla cells and modulates hair folliculogenesis [69].

#### 3.4.4. Hair Follicle Maturation

Subsequent hair follicle differentiation and maturation largely depends on signals from the dermal papilla. Master signal pathways in early stage of hair follicle morphogenesis, such as Wnt and BMP signaling, still function during the maturation stage. Wnt activity in the matrix cells is important for hair shaft differentiation [70], while Wnt activity in the dermal papilla is responsible for its hair inductive ability and regulates hair morphogenesis [71,72]. BMP activity in the epidermal compartment is crucial for matrix cell differentiation since ablation of BMP receptors and overexpression of BMP inhibitor both prevent hair shaft maturation [73–75]. BMP activity in the dermal papilla is also required for its hair inductive properties [76]. In addition, FGF signaling and Notch signaling are also implicated in hair follicle maturation.

**Figure 2:**
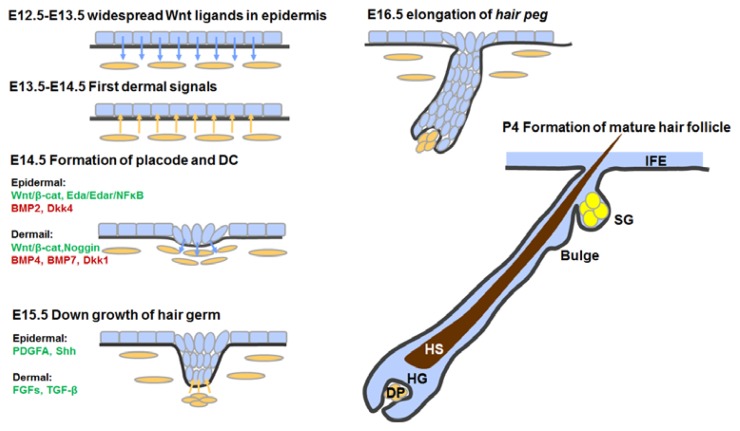
Events and key regulators during hair follicle morphogenesis. DC, dermal condensate; DP, dermal papilla; HG, hair germ; HS, hair shaft; SG, sebaceous gland; IFE, interfollicular epidermis; β-cat, β-catenin.

The main stages and events during hair follicle morphogenesis are outlined in Figure 2. From above, we can see that hair follicle morphogenesis greatly relies upon close and regulated dermal-epidermal interactions. It should be noted that different types of hair follicles require distinct signaling patterns. Specific signals also contribute to the variety of hair follicle types. Besides, the dermis also requires epidermal signals to differentiate and to be specified. Sustained activation of β-catenin in the epidermis can reprogram the adult dermis into a neonatal state, and the reprogrammed dermis could in turn induce ectopic hair follicle formation [77]. In conclusion, hair follicle morphogenesis is a series of interactions and joint development of the epidermal and dermal compartments.

### 3.5. Development of Sweat Glands

In humans, the sweat glands start to develop during Week 13–14 of gestation and mature at about Week 24 [78]. In mouse embryos, sweat gland germs emerge as invaginations of epidermal basal cells at E17.5, shortly before birth. During postnatal day P1–P5, sweat gland germs develop into single long ducts extending deeply into the dermis, with coiled glands at the tip [79]. Formation of sweat gland germs depends on the activation of epidermal Eda signaling, just as in hair placode formation. However, in the hair placodes, Eda signaling is activated as a downstream event of Wnt signaling, whereas Wnt signaling defects in Lef-1 deficient mice do not harm sweat glands [80], suggesting that some other unknown signal precedes the activation of Eda signaling in sweat gland formation. Like in hair placode down-growth, Eda signaling activates and up-regulates the downstream Shh pathway in initial sweat gland development. Shh signaling activity sustains to the coiling stage and was down-regulated when sweat gland development is complete. The development and maintenance of the coiled secretive portion was suggested to depend on Fox genes, including Foxa1 and Foxi1, both not seen in hair follicles [79].

## 4. Stem Cells in Epidermal Development and Homeostasis

Recent studies have revealed that there are several types of stem cells residing in different niches in mammalian epidermis. These stem cells, distinct in marker expression patterns and growth properties, contribute differently to the homeostasis of diverse epidermal compartments. With recent progress in lineage tracing techniques, some light has been shed on the developmental origins and exact differentiation tracks of certain epidermal stem or progenitor cells. In this part, we will briefly review the general properties of diverse epidermal stem cell populations, attempt to clarify their specification and contribution in skin development and homeostasis, and discuss some master signals that regulate their self-renewal and differentiation.

### 4.1. Epidermal Stem Cells in the Interfollicular Epidermis (IFE)

It has been agreed for years that IFE stem cells reside in the basal layer and they are responsible for maintaining the life-time renewal of the IFE. There are two different models describing the proliferation and differentiation patterns of IFE stem cells in mature epidermis. The long-held epidermal proliferative unit (EPU) model describes epidermal keratinocytes organized into a column-like structure. Within this column, one of the basal cells is the stem cell and the other basal cells are transit-amplifying (TA) cells which continue to differentiate and move outwards to generate all the suprabasal cells [81,82]. This model is supported by the fact that the basal layer cells are heterogeneous with respect to their proliferative properties and the expression level of integrins [83], which, as we mentioned in the previous section, are important for the cells to adhere to the basement membrane and maintain their proliferative ability and undifferentiated properties. It is also supported by the observation that the cells with the highest integrin-β1 level generate the largest clones (holoclones) with long-term self-renewal capacity *in vitro* [83]. Therefore, integrins are used for years as markers to enrich epidermal stem cells *in vitro*. A second model comes from recent *in vivo* studies in which asymmetrical divisions are found in basal layer cells [26,84,85]. In this asymmetrical division model, the stem cells divide to generate a self-renewing daughter stem cell and a daughter cell committed to differentiate, through asymmetrical distribution of key factors, whether the division occurs laterally or perpendicularly to the basement membrane [86]. In such a model, there is no intermediate state as TA cells. Although these two models are not necessarily mutually exclusive, they do lead to distinct answers to key questions regarding the number of IFE stem cells and the exact location of their niche within the basal layer. A recent study combining clonal analysis, lineage tracing experiments and proliferation kinetic measurements in mouse IFE provided a reconciliation of these two theories. In adult mouse, hierarchical organized slow cycling stem cells and rapidly cycling committed progenitors co-exist in the IFE and function differently during IFE homeostasis and repair [87]. The balance between their proliferation and differentiation is suggested to be achieved through stochastic fate choice [87].

Some key regulators for IFE stem cell maintenance have been identified. The basement membrane plays a crucial integral role in controlling IFE stem cells to maintain homeostasis under different conditions, through its mechano-physical properties, ECM, as well as growth factors. The integrin/FAK signaling, TGF-β signaling and EGF signaling are involved in this complex regulation network. Besides, DNp63, the master regulator of epidermal development, is suggested by some groups to function in maintaining the self-renewal of IFE stem cells [19]. This particular role of DNp63 is argued by others [7]. Nonetheless, it is generally agreed that DNp63 is preferentially expressed in basal cells and is pivotal for keeping their proliferative properties [7,16,88]. Since the question about the basal IFE stem cells and TA cells is unsolved, the exact roles of p63 in IFE stem cells and TA cells also need further exploration.

Almost all the studies about IFE stem cells are performed in mature skin. What is the case in embryonic epidermis is even more elusive. It is still largely unknown when and how IFE stem cells are specified, and what signals control their behaviors. Our lab has made some effort to study the fetal human epidermal stem cells. We initially enriched these cells using fluorescence-activated cell sorting (FACS) based on their high level of integrin-β1 expression [89], and then we replaced this method with a combination of fast adhesiveness to type IV collagen and cell size sorting, which was more effective and reliable [90]. With these enriched putative fetal epidermal stem cells, we revealed the effects of Shh and Wnt signaling in promoting their *in vitro* proliferation [91,92]. The components of the two pathways are also detected in fetal human basal layer. Whether they play a similar role in human epidermal development is yet to be determined.

### 4.2. Hair Follicle Stem Cells in the Classic Bulge Niche and the Hair Germ

A hair follicle undergoes continuous cycles of degenerations and regenerations throughout life, during which lower portion of the hair follicle is renewed, leaving the upper portion as a permanent part. The cyclic regeneration of hair follicles is maintained by hair follicle stem cells (HFSCs). HFSCs were initially identified in mice as slow-cycling label-retaining cells (LRCs) within the bulge region which locates at the lowest permanent portion of a hair follicle [93]. Grafting experiments demonstrated that these bulge cells can regenerate the epidermis and hair follicles [94,95]. According to their location, bulge cell surface markers CD34 and integrin-α6 were designated as candidate HFSC markers and used for many years to isolate HFSCs. During the past few years, using cell lineage tracing in adult mice, HFSCs expressing distinct markers (Sox9 [96], K15 [94,97], K19 [98], Lgr5 [99] and gli1 [100], *et al*.) have been identified in the bulge area, as well as in the hair germ. Our group isolated and expanded bulge stem cells from rat vibrissa hair follicles [101]. These cells support the hair follicle regeneration throughout adult homeostasis, and contain both quiescent stem cells and stem cells that are actively dividing, as demonstrated in several other tissues. The detailed contribution of each HFSC subpopulation to the hair follicle is reviewed elsewhere [102].

At the start of each hair cycle, HFSCs must be activated to generate the new hair follicle. HFSC activation has two steps, with the hair germ stem cells responding to the dermal papilla signals and motivated first, followed by the bulge stem cell activation [103]. It is also indicated that the hair germ cells are derived from the bulge stem cells [103]. Activated bulge stem cells migrate out of the bulge, proliferate, differentiate and move downward the hair follicle. These activated HFSCs can maintain their stem-ness and home back to the bulge niche until they reach a “point of no return”. Therefore, the bulge niche seems only required to maintain the quiescent state of HFSCs, but not necessary for the “stem-ness” of HFSCs. In order to support both homeostasis and regeneration, HFSCs are dynamically regulated to keep a balance between quiescence and activation, the violation of which can result in diseases or tumors. BMP signaling keeps HFSCs in a quiescent state [104] and Wnt/β-catenin signaling is important for their activation [105]. These two master signaling pathways function together to regulate HFSC state during different phases of the hair cycle [106]. In addition, TGF-β signals also control HFSC activation and maintenance [107,108] and disruption of Smad4 leads to HFSC depletion [109]. Alterations in physiological condition, such as hormone fluctuations, can influence the hair cycle mainly through these master regulators [110].

Under physiological conditions, bulge HFSCs only contribute to the hair follicle lineages [94,98,99]. However, upon wounding, they also participate in the regeneration of the IFE [111,112]. This suggests that the IFE and hair follicles are normally maintained by different sources of stem cells in adult skin. Actually, the IFE and hair follicle lineages are separated as early as the hair placode formation. This is indicated by embryonic lineage tracing studies, in which all the hair follicle cells are derived from Shh^+^[113] and Sox9^+^[96] hair follicle progenitors, while none of the IFE cells has such an origin. Although neither obvious bulge niche architecture nor bulge cell markers occur until about P20 in mice, slow-cycling HFSCs are already specified and function earlier during hair follicle morphogenesis (by P2) and later give rise to adult HFSCs [96]. The specification of these embryonic HFSCs requires Sox9. Sox9 deficient hair follicles cannot form the outer root sheath (ORS), upon which the future bulge niche will be built, and thus fail to establish the HFSC compartments [96,114].

### 4.3. Stem Cells in the Isthmus, Sebaceous Gland and Infundibulum

While the cycling lower part of a hair follicle is maintained by bulge stem cells, the homeostasis of the upper hair follicle, on the other hand, depends on other stem cells. The upper segment of a hair follicle (above the bulge) consists of 3 parts: the isthmus which is a narrow continuation of the bulge, the sebaceous gland with an opening in the isthmus, and the uppermost infundibulum which is a joint part of the hair follicle and IFE. Recent studies reported several groups of stem cells in the upper hair follicle. Lgr6^+^ stem cells reside directly above the bulge in mature skin [115]. MTS24/Plet1 expressing stem cells are found in the isthmus between the sebaceous gland and the bulge [116]. Lrig1^+^ stem cells, located in the upper isthmus and the lower infundibulum, are largely quiescent [117]. These stem cell populations barely express classical bulge stem cells markers such as CD34 and K15, but are all able to give rise to the hair follicle and IFE in skin reconstitution assays. However, under physiological conditions, they mainly contribute to only sebaceous gland and IFE. These stem cell populations show partial positional overlap with one another and have different gene expression profiles, indicating their distinct nature. In addition, a group of Blimp1^+^ unipotent progenitor cells that only contribute to the sebaceous gland were discovered residing at the opening of the sebaceous gland [118].

During hair follicle development, Lgr6 is expressed in the earliest embryonic hair placodes, just like Shh and Sox9. However, the difference is that the progeny of embryonic Lgr6^+^ cells covers all the epidermal cell lineages [115]. Similarly, the Lrig1^+^ cell population is as well specified early in hair follicle development, coinciding with the formation of presumptive bulge HFSCs [96,117]. In addition, Lrig1^+^ progenitors also give rise to the sebaceous gland by asymmetric cell division, while MTS24^+^ progenitors seem dispensable for sebaceous gland formation [119]. It is shown that distinct stem and progenitor populations are established at different time points of hair follicle development [119]. However, the exact relationships between the embryonic Shh^+^, Sox9^+^, Lgr6^+^ and Lrig1^+^ populations still needs to be clarified. How these embryonic progenitor cells evolve and persist as adult stem cell populations is also a very interesting question.

### 4.4. Sweat Gland Stem Cells

Unlike the IFE and hair follicle, sweat glands show little cellular regeneration. The existence of sweat gland stem cells remained largely unexplored until very recently. Using lineage tracing in mouse paw skin, which is the only sweat gland-containing skin in mice, Lu *et al*. identified multipotent sweat gland progenitors in development, as well as distinct unipotent stem cell populations within both sweat ducts and the coiled glands in adult skin [120].

The sweat gland is comprised of a sweat duct and a coiled gland. The sweat duct contains a basal layer (K5/K14^+^) and a suprabasal layer (K8/18^+^), which respectively become the myoepithelial cell layer (K5/K14^+^ and SMA^+^) and the luminal cell layer (K8/K18^+^ and K19^+^) in the coiled gland. During development, the sweat duct and coiled gland are derived from K5/K14^+^ epidermal progenitors in the basal layer. A common multipotent “sweat bud progenitor” generates the sweat duct containing K14^+^ basal progenitors and K14^+^ and K18^+^ suprabasal progenitors, both of which are still multipotent at this time point. During sweat duct maturation, these progenitors expand separately within their layers and then produce the myoepithelial cells and the luminal cells of the coiled gland, respectively. During this process, these progenitors gradually lose their potential and become unipotent. In adult sweat gland, the unipotent ductal stem cells reside in the basal layer and they give rise to the basal and suprabasal cells in the duct. On the other hand, the glandular myoepithelial stem cells and the luminal stem cells only contribute to their own lineages, respectively [120].

**Figure 3:**
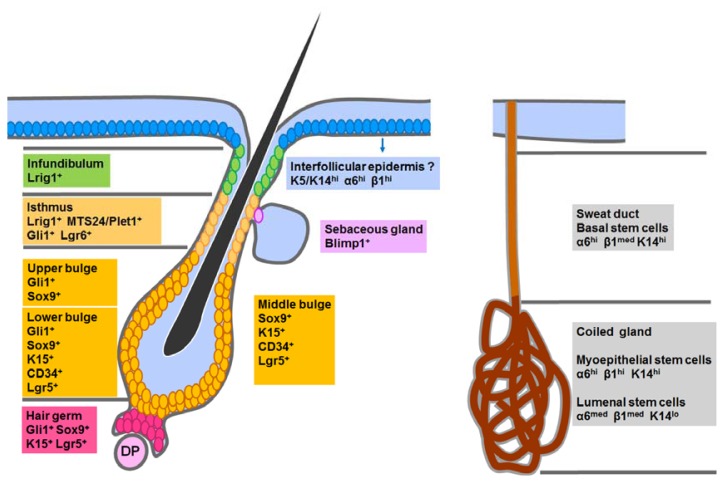
Different epidermal stem cell populations residing in the IFE, telogen HF and the sweat gland. Different markers for each stem cell population are summarized. +, positive; hi, high; med, medium; lo, low; DP, dermal papilla.

In summary, there are several different stem cells residing in distinct niches within epidermis, as shown in Figure 3. Although different, these stem cell populations do share some similarities. Almost all of these stem cells (except the sweat gland luminal stem cell) reside in the basal layer, clinging to the basement membrane and express high levels K5, K14 and integrins [86]. Besides, the behaviors of these stem cells are similar to some extent and there are common principles regulating their proliferation and differentiation. Another important common feature of the epidermal stem cell populations is their potential multipotency. Although a certain stem cell population normally generate limited cell lineages during homeostasis, they tend to give rise to all epidermal lineages, including IFE, hair follicles and sebaceous glands, under regenerative conditions created either by wounding, transplantation together with embryonic mesenchyme, or over-activation of certain signals [94,111,112,121]. These observations also indicate the significance of the underlying dermis in directing the differentiation potential of epidermal stem cells, just as in the epidermal developmental process.

## 5. Derivation of Epidermal Lineages from Pluripotent Stem Cells *In Vitro*

According to the roles of key regulators and signaling pathways during embryonic epidermal development, researchers started to seek for an *in vitro* model for epidermal lineage differentiation. Because of their limitless self-renewal and extensive differentiation potential, pluripotent stem cells are induced to differentiate into various specialized cell types for *in vitro* studies of lineage development, and for cellular and tissue-based therapies. During the last decade, progress has been made to derive epidermal cells and structures from embryonic stem cells (ES cells) and induced-pluripotent stem cells (iPS cells).

### 5.1. From ES Cells to Epidermal Lineages

Mouse ES cells have been successfully differentiated into cell populations committed to epidermal lineages [122–125]. Inspired by these studies, human ES cells have also been used to generate epidermal cells, which offers an even more useful *in vitro* system to study mechanisms underlying a variety of human skin disorders. Remarkably, the pluristratified epidermis reconstituted from keratinocyte progeny of human ES cells provided accessible temporary skin substitutes for patients with large burns waiting for grafting [126].

Following the physiological pathway toward epidermal differentiation *in vivo*, signaling factors, such as BMP4 and all-trans retinoic acid (RA), and ECM components have been used as inducers or enhancers for epidermal differentiation from ES cells *in vitro*. The generation of keratinocyte precursors can be regulated by ECM and soluble chemical factors in the induction medium. In the following paragraphs, we shall discuss the roles of BMP4, RA and ECM components in epidermal lineage differentiation of ES cells *in vitro*.

#### 5.1.1. BMP4

*In vivo*, BMP4 is a potent epidermal inducer and neural inhibitor at the time of ectodermal fate determination [12]. *In vitro*, during mouse ES cell differentiation, BMP4, in concert with somatic feeder cells [127], could bias the ectodermal binary decision towards the epidermal fate. It is also figured out that there is a BMP4-sensitive time window during mouse ES cell differentiation along epidermal fate [128]. During mouse ES cell differentiation, BMP4 treatment could induce the apoptosis of Sox1^+^ neural progenitors and promote epidermal commitment by down-regulating Smad-6 [129]. In addition, AP-2γ, a member of the activator protein (AP)-2 family, is up-regulated by BMP4, and could partially mediate the function of BMP4 [130]. During human ES cell differentiation, expression of early markers of neuroectoderm, such as Sox1 and PAX6 [131,132], is also inhibited by BMP4 [133]. After 40 days of culture with 3T3 feeder cells, differentiated human ES cells acquired phenotypic keratinocyte characteristics in medium supplemented with ascorbic acid and BMP4. After the long-term succession, full differentiation of basal keratinocytes, which can release molecular components for epidermal-dermal junction, has been achieved and these keratinocyte progeny can form a pluristratified epidermis both *in vitro* and *in vivo* [126].

#### 5.1.2. RA

Retinoids, especially all-trans-retinoid acid (RA), are essential for embryonic patterning and development of chordates. Vitamin A, metabolized from RA, plays an important role in epithelial-mesenchymal and epithelial-epithelial interactions [134–136]. *In vitro*, the function of RA during ES cell and keratinocyte differentiation is stage-dependent. First, RA treatment could promote the expression of ectoderm markers during spontaneous differentiation of human ES cells in 3D scaffold culture [137]. Second, ectodermal derivatives of ES cells acquire epithelial fates in response to RA in a stage-specific manner [133]. Third, this peptide growth factor is also reported capable of up-regulating the expression of p63, a primary driver of epithelial cell enrichment [138], in human keratinocytes, which in turn could promote terminal differentiation of keratinocytes [139]. Therefore, RA has a promoting effect on ES cell differentiation along the keratinocyte lineage. Because ectodermal derivatives from ES cells abandon neuroepithelial fate in response to BMP4, the distinct but synergic roles of RA and BMP4 provide an efficient system to direct epidermal differentiation of human ES cells [133].

#### 5.1.3. ECM Components

Given that every step of embryonic epidermal development largely depends on dermal mesenchyme and ECM, different ECM components are also used to facilitate *in vitro* ES cell differentiation to epidermal lineages. Type I collagen was reported to support the long-term maintenance of a homogeneous population of K8/K18^+^ ectodermal cells derived from human ES cells, and these ectodermal cells are capable of further differentiating into K5/K14^+^ epidermal-like cells with exogenous DNp63 expression [140]. When undifferentiated mouse ES cells were directly seeded on plates coated with matrix secreted by human mesenchymal fibroblasts, enrichment of K14^+^ colonies was clearly observed, together with LIF removal and BMP4 supplement in the induction medium [123]. When mouse ES cells were differentiated via embryoid body (EB) formation, type I collagen and the basement membrane matrix, Matrigel, could both promote the cell migration and epithelialization of EBs [124]. In addition, after human ES cell-derived EBs attached on type I collagen-coated dish and were cultured in defined keratinocytes serum-free medium (DKSFM), cells that firstly expressed p63 then expressed K14 were seen migrate out of the cores of EBs [141]. However, it is reported that Matrigel could not promote human ES cell differentiation to epidermal lineages as they do in mouse ES cells [124], while both type I collagen and gelatin could [141].

### 5.2. From iPS Cells to Epidermal Lineages

The potential use of ES cells for cell and gene therapies is limited by ethical problems and difficulties in genetic manipulation. In addition, ES cell-derived keratinocytes are not able to provide personalized therapies to patients with varied skin disorders. Therefore, the generation of iPS cells offers an attractive system that can overcome concerns about ES cells mentioned above.

iPS cells were initially generated by viral vector-mediated overexpression of pluripotency factors (Oct4, Sox2, Klf4, and c-Myc) in fibroblasts [142,143]. Considering the risk of insertional mutagenesis of viral vectors and spontaneous transgene reactivation, alternative gene-delivering vectors have been developed in recent years for therapeutic purposes, such as transposons [144], proteins [145], microRNAs [146], messenger RNAs [147], and so on. Because of the easy accessibility of somatic cells used for iPS cell generation, the therapeutic potential of iPS cells with ES cell-like properties is enormous in patient-specific tissue repair and drug evaluation. For instance, iPS cells generated from fibroblasts of Alzheimer’s disease patents have been successfully differentiated into cortical neurons which could be applied for drug test [148,149].

The protocol for keratinocyte differentiation from iPS cells was firstly established in mice [150]. K14^+^ cells were generated through EB formation, sequential supplement with RA and BMP4, and further enrichment on type IV collagen-coated dishes. Keratinocytes derived in this way could not only initiate terminal differentiation in response to high calcium concentration *in vitro*, but also regenerate fully differentiated epidermis, hair follicles, and sebaceous glands *in vivo* [150]. Recently, epidermal differentiation from human iPS cells was also achieved. Tolar *et al*. demonstrated the derivation of patient-specific iPS cells from two patients with recessive dystrophic epidermolysis bullosa (RDEB), a disease characterized by skin fragility, chronic blistering, and erosions caused by mutation in COL7A1 gene [151]. Later, Itoh *et al.* showed that RDEB-iPS cells could differentiate into keratinocytes, and subsequently, into 3D skin equivalents with expression of markers for terminal epidermal differentiation [152]. These patient-specific iPS cell-derived keratinocytes provide an *in vitro* model to study such skin diseases. With gene correction, they would also present an opportunity for the future systemic treatment of these diseases.

### 5.3. Comments on These *In Vitro* Studies

Although progress has been made, there are some major stumbling blocks in the derivation of epidermal lineages from pluripotent stem cells.

Firstly, the long duration of differentiation process *in vitro* severely increases the diversity of daughter cell population. Appearance of heterogeneous cell populations during induction is unavoidable and could lead to tumor formation when these cells were transplanted *in vivo*. Therefore, the long-playing induction process could decrease the global differentiation efficiency, augment the difficulties to enrich epidermal lineage cells of interest, and lead to spontaneous differentiation.

Secondly, the K14^+^ cells that are differentiated from pluripotent stem cells are not equivalent to normal epidermal stem cells. On the one hand, they cannot proliferate stably *in vitro* compared with primary epidermal stem cells. On the other hand, terminal differentiation of these *in vitro*-derived K14^+^ cells only occurs infrequently, resulting in inadequate epidermal functions [124].

Thirdly, these ES cell or iPS cell derivatives exhibit low proliferative capacity. Methods to extend the lifespan of these cultures need to be explored. Furthermore, the self-renewal capacity of these cells after long-term transplantation should be estimated in proper microenvironment *in vivo*. Compatibility and genomic stability of these progeny of pluripotent stem cells and their derivative structures must be ensured.

Fourthly, step-by-step ES cell differentiation along epidermal lineages is more convincing than one-step uninterrupted differentiation. As previously stated, epidermal development *in vivo* is a multistage process including epidermal specification, commitment, stratification and terminal maturation, during which surface ectodermal precursors, epidermal progenitors and terminally differentiated keratinocytes occur at different time points. One-step induction results in unfavorable absence of key molecular markers and ECM components that could directly and effectively enhance specific differentiation along the epidermal lineages.

Last but not least, the microenvironment of epidermal embryogenesis cannot be neglected, especially signaling inducer and/or inhibitors from the sub-epidermal mesenchyme. Besides the well-known BMP4 and RA, it is necessary to apply more inducers or inhibitors, which could restraint other lineages than the epidermal lineages, to an appropriate *in vitro* model system.

## 6. Concluding Remarks

During the preparation of this review, we came to realize that the whole knowledge about epidermal development and epidermal stem cells is like a big treasure map. While some parts of the map are depicted in detail, some parts are too vague to zoom in and some parts are even still missing. Notably, early epidermal morphogenesis is largely a gap. What mesenchymal signals induce the surface ectoderm to adopt an epidermal fate and initiate stratification? How do these signals function? How is the development of sebaceous glands and sweat glands regulated? Are there only a small portion of stem cells in the basal layer of IFE or all of the basal cells are stem cells? What are the exact relationships between distinct stem cell populations during development? To what degree are behaviors of these epidermal stem cells dependent upon their distinct niche environment? There are still so many questions to be addressed. Importantly, most studies on epidermal development are performed in mice, in which genetic manipulations are possible. However, human skin differs greatly from the mouse skin in terms of epidermal thickness, hair clothing, sweat gland distribution, and so forth. Are these mechanisms in mice still suitable in humans? What human-specific mechanisms are responsible for skin disorders such as alopecia? To answer these questions will not only help us study the general rules about development and stem cell regulation in other tissues, but also contribute greatly to the understanding and potential treatment of skin diseases and tumors.
